# Impact of Hospital Volume on In-Hospital Cardiac Arrest Mortality: Trends Before and During the COVID-19 Pandemic

**DOI:** 10.7759/cureus.87743

**Published:** 2025-07-11

**Authors:** Chau Doan Nguyen, Viet Nghi Tran, Sayma Yaqub, Huan Dat Pham, Thien Vo

**Affiliations:** 1 Internal Medicine, Texas Tech University Health Sciences Center, Amarillo, USA; 2 Internal Medicine, Weiss Memorial Hospital, Chicago, USA; 3 Internal Medicine, Conemaugh Memorial Medical Center, Johnstown, USA; 4 Pulmonology and Critical Care, Texas Tech University Health Sciences Center, Amarillo, USA

**Keywords:** covid-19 pandemic, hospital volume, in-hospital cardiac arrest, mortality outcomes, propensity score matching

## Abstract

Background

Hospital volume is frequently associated with clinical outcomes in patients with critical illnesses. This study aims to evaluate the impact of hospital volume on in-hospital mortality among patients with in-hospital cardiac arrest (IHCA) during the pre-pandemic period (2017-2019) and the COVID-19 pandemic era (2020-2021), using data from the Nationwide Inpatient Sample (NIS) database.

Methods

We conducted a retrospective analysis of 304,030 IHCA admissions (2017-2019) and 287,695 admissions (2020-2021) using the NIS database. Hospitals were categorized by annual IHCA volume into three groups: low (≤33rd percentile), medium (33rd-67th percentile), and high (≥67th percentile). Survey-weighted logistic regression was used to assess the association between hospital volume and in-hospital mortality, adjusting for demographics, comorbidities, hospital characteristics, and the Elixhauser index. Propensity score matching was used for pairwise comparisons across volume groups.

Results

During the pre-pandemic period (2017-2019), high-volume hospitals were associated with significantly higher in-hospital mortality than medium-volume hospitals before propensity score matching (adjusted odds ratio (OR): 1.100; 95% CI: 1.035-1.169; p = 0.02). After matching, this association persisted, with high-volume centers showing a modest but statistically significant increase in mortality (OR: 1.075; 95% CI: 1.001-1.154; p = 0.046). In the pandemic period (2020-2021), high-volume hospitals remained associated with higher IHCA mortality, with significantly elevated odds of death compared to both low-volume (OR: 1.239; 95% CI: 1.101-1.394; p < 0.001) and medium-volume hospitals (OR: 1.115; 95% CI: 1.026-1.211; p = 0.01) after matching. When stratified by COVID-19 status, high-volume hospitals in the non-COVID-19 subgroup continued to show significantly higher mortality rates than medium-volume hospitals after adjustment and matching (OR: 1.097; 95% CI: 1.009-1.192; p = 0.003). However, among COVID-19-positive patients, no statistically significant associations were found between hospital volume and in-hospital mortality after matching (medium vs. low: OR 1.125; 95% CI: 0.856-1.480; p=0.398; high vs. low: OR 1.387; 95% CI: 0.942-2.042; p = 0.098; high vs. medium: OR 1.116; 95% CI: 0.883-1.411; p=0.357).

Conclusion

Hospital volume was associated with in-hospital mortality, varying by pandemic period and patient subgroup. Before the pandemic, high-volume hospitals had a modestly higher mortality rate, a pattern that persisted among non-COVID-19 patients during the pandemic. No significant association emerged for COVID-positive patients, suggesting that system-level strain, rather than viral effects, was the primary driver. These findings highlight the need for surge-adaptive protocols to maintain IHCA care quality during periods of operational stress.

## Introduction

The American Heart Association (AHA) defines cardiac arrest as the sudden cessation of cardiac activity, resulting in unresponsiveness, apnea, and pulselessness. Cardiac arrest can occur out-of-hospital (OHCA) or in-hospital (IHCA), with the latter affecting over 290,000 adults annually in the United States [1]. Unlike OHCA, IHCA events are often preceded by warning signs and may be preventable through early detection and intervention. Advances in resuscitation have improved IHCA survival rates from 13.7% in 2000 to 26.7% in 2019 [2]. However, this progress stalled during the COVID-19 pandemic, when survival rates dropped to 21% in 2020 [3], below the AHA's goal of greater than 24% by 2030 [4]. Therefore, understanding and addressing the factors that limit further improvement is essential.

One area of growing interest is the relationship between hospital volume and cardiac arrest survival. It is plausible that hospitals managing a higher number of IHCA cases may develop more experienced resuscitation teams and streamlined code protocols, leading to faster recognition, timely CPR, and better post-arrest care [5]. However, findings regarding the association between hospital volume and cardiac arrest outcomes have been inconsistent. A national South Korean cohort study of 337,042 cardiac arrest admissions compared outcomes across tertiary, general, and community hospitals. It found significantly lower long-term mortality in tertiary centers, likely due to better access to specialized post-arrest care, even after adjusting for clinical and procedural variables [6]. However, a large prospective study of 125,082 IHCA cases found that hospitals with the highest CPR volumes did not achieve better outcomes than lower-volume centers. In fact, lower-volume hospitals sometimes performed better [7]. This study adjusted for patient comorbidities and hospital characteristics, suggesting that high case volume alone may not confer a survival advantage.

Importantly, the COVID-19 pandemic introduced unprecedented strain on healthcare systems, including staffing shortages, ICU overcrowding, and disrupted workflows. These factors may have altered the volume-outcome relationship by amplifying the operational challenges faced by high-volume centers. Therefore, examining hospital volume separately during the pre-pandemic and pandemic periods may yield new insights into how systemic pressure impacts IHCA outcomes.

We hypothesize that hospital volume is associated with IHCA mortality, but this relationship may vary depending on external stressors. Specifically, while higher volume may reflect greater experience, it may also introduce risks such as resource strain, delayed responses, or staff fatigue. Our objective was to assess whether hospital IHCA volume was associated with in-hospital mortality and whether this association changed before and during the COVID-19 pandemic.

## Materials and methods

Data source

This study used data from the Nationwide Inpatient Sample (NIS) database, the largest all-payer database of hospital discharges in the United States. The NIS captures approximately 20% of all inpatient discharges nationwide and is designed using a stratified sampling method that includes discharge weights to produce nationally representative estimates of hospital care [8].

Patient population

We included patients aged 18 years and older with a secondary diagnosis of cardiac arrest, identified using ICD-10 codes I46, I97.12, and I97.71. This approach was used to focus on IHCA, as patients with OHCA are often admitted with a primary diagnosis of cardiac arrest. To ensure accurate outcome attribution, we excluded patients with documented do-not-resuscitate (DNR) orders and those who were transferred out, as their in-hospital mortality could not be reliably assigned to the originating hospital.

Variables

We accounted for several covariates and independent risk factors associated with in-hospital mortality. At the patient level, we included demographic variables (age, race, sex, and socioeconomic status, indicated by insurance type and median household income) and pre-existing comorbidities such as chronic ischemic heart disease (CIHD), heart failure (HF), cardiomyopathy, chronic lung disease, diabetes mellitus, obesity, chronic kidney disease (CKD), hypertension, and hyperlipidemia. These covariates have been consistently shown to be associated with in-hospital mortality in patients with cardiac arrest or critical illness in prior studies [9]. Comorbidities were identified using ICD-10 codes, and the comorbidity burden was quantified using the Elixhauser Comorbidity Index (ECI), with higher scores indicating a greater burden. We selected the ECI because it has been extensively validated for use in administrative databases, such as the NIS, and has demonstrated superior performance over other indices (e.g., Charlson Comorbidity Index) in predicting in-hospital mortality and healthcare utilization outcomes in large inpatient cohorts [10].

At the hospital level, we included variables defined according to NIS criteria: location/teaching status (urban-teaching, urban non-teaching or rural non-teaching), based on Core-Based Statistical Area designation and teaching hospital characteristics such as residency programs or intern/resident-to-bed ratios; bed size (small, medium, or large), classified relative to hospital region, location, and teaching status; geographic region (Northeast, Midwest, South, or West), as defined by the U.S. Census Bureau; and ownership type (government nonfederal, private nonprofit, or private for-profit). The outcome variable was in-hospital mortality, defined as death occurring during the index hospitalization, based on discharge disposition.

Statistical analysis

Hospitals were categorized into low-, medium-, and high-volume groups based on annual IHCA case volume, using the 33rd and 67th percentile cutoffs to define tertiles. This percentile-based approach ensures approximately equal group sizes, thereby enhancing statistical power and improving model stability. In the absence of a universally accepted hospital volume threshold for IHCA, tertile stratification provides a non-arbitrary method for evaluating volume-outcome relationships. Similar approaches have been used in previous literature; for example, Kontos et al. stratified hospitals into tertiles based on the proportion of myocardial infarction cases presenting with cardiac arrest [11], and Akintoye et al. categorized hospital volume into quartiles when analyzing outcomes of IHCA [7]. Hospitals below the 33rd percentile were classified as low-volume, those between the 33rd and 67th percentiles as medium-volume, and those above the 67th percentile as high-volume. The corresponding volume thresholds were as follows: for the pre-pandemic period, low (≤95 cases), medium (96-185 cases), and high (≥186 cases); for the pandemic period, low (≤130 cases), medium (131-255 cases), and high (≥256 cases). Baseline characteristics across volume groups were compared using the chi-square test for categorical variables and analysis of variance (ANOVA) for continuous variables. However, no post hoc tests were conducted on these descriptive statistics, as the primary analysis used adjusted models and pairwise propensity score matching.

To assess the association between hospital volume and in-hospital mortality, we used a multivariable logistic regression model. Covariates were selected based on clinical relevance and established predictors of survival from prior literature, rather than relying solely on univariate significance testing. Variables included age, sex, race, ECI, cardiovascular and pulmonary comorbidities, metabolic diseases, insurance type, income quartile, hospital bed size, teaching status, geographic region, and hospital location. This approach was designed to minimize residual confounding and improve model robustness. The significance of categorical variables was assessed using omnibus tests, including the Wald test. All analyses incorporated the NIS's complex survey design, accounting for stratification, clustering, and discharge weights, using STATA's "svy" commands. This ensured accurate variance estimation and allowed for the generation of nationally representative estimates.

All statistical analyses were conducted using STATA version 18 (StataCorp LLC, College Station, TX, US) [12], with a two-tailed significance level set at 0.05.

We conducted an annual trend analysis from 2017 to 2021 and subsequently grouped the data into two time periods (2017-2019 and 2020-2021) to compare and evaluate the overall effect of hospital volume on in-hospital mortality among patients with cardiac arrest. To improve the accuracy of comparisons, we applied propensity score matching and conducted pairwise analyses between hospital volume categories: medium vs. low volume, high vs. low volume, and high vs. medium volume. We used 1:1 nearest-neighbor matching without replacement, with a caliper of 0.2 times the standard deviation of the logit of the propensity score. Matching variables included age, sex, race, insurance type, income quartile, hospital region, teaching status, bed size, individual comorbidities, and the ECI. ​​The sample sizes remained balanced between matched groups, supporting the validity of the comparisons.

## Results

Between 2017 and 2019, our study included a total of 304,030 IHCA cases: 103,345 in 2017, 101,100 in 2018, and 99,585 in 2019. During the COVID-19 pandemic, IHCA cases increased, with 130,805 cases in 2020 and 156,890 in 2021, totaling 287,695 cases for the pandemic period. The number of IHCA in-hospital deaths was 69,845 (67.58%) in 2017, 65,860 (65.14%) in 2018, 62,705 (62.97%) in 2019, 91,435 (69.90%) in 2020, and 110,430 (70.39%) in 2021.

Year 2017

Hospital volume was not associated with significant differences in in-hospital mortality. Compared to low-volume hospitals, medium-volume centers had an odds ratio (OR) of 0.919 (95% CI: 0.829-1.020), and high-volume centers had an OR of 1.035 (95% CI: 0.911-1.176).

Year 2018

No significant association was observed between hospital volume and mortality. Medium-volume hospitals had an OR of 0.982 (95% CI: 0.885-1.090), while high-volume hospitals had an OR of 1.011 (95% CI: 0.885-1.154), compared to low-volume hospitals.

Year 2019

Hospital volume remained a non-significant predictor of mortality. The OR for medium-volume hospitals was 0.984 (95% CI: 0.890-1.089), and for high-volume hospitals, it was 1.080 (95% CI: 0.939-1.242), compared to low-volume hospitals.

Year 2020

A marked shift was observed during the first year of the pandemic. Medium-volume hospitals had higher mortality with an OR of 1.126 (95% CI: 1.015-1.250), and high-volume hospitals also showed increased mortality (OR: 1.319; 95% CI: 1.164-1.494) relative to low-volume hospitals.

Year 2021

This pattern persisted in 2021. Medium-volume hospitals had an OR of 1.190 (95% CI: 1.073-1.320), and high-volume hospitals had an OR of 1.226 (95% CI: 1.085-1.386), both indicating higher mortality compared to low-volume institutions.

We observed a trend wherein higher hospital volume was associated with increased IHCA in-hospital mortality during 2020 and 2021, particularly in medium- and high-volume centers. To better assess this shift, we grouped all IHCA cases from 2017 to 2019 and from 2020 to 2021. To further evaluate the impact of COVID-19, IHCA admissions from 2020 and 2021 were stratified into two subgroups: COVID-positive and non-COVID. We initially conducted the analysis, then applied propensity score matching and re-ran the models to improve comparability across hospital volume groups.

Among IHCA patients from 2017 to 2019, the mean age was 62.02 years in high-volume hospitals, 63.14 years in medium-volume hospitals, and 63.50 years in low-volume hospitals. Female patients accounted for approximately 39.9% to 41.6% of the cohort. White patients constituted the largest racial group (60.9%-68.01%), followed by Black, Hispanic, Asian, and Native American patients. The prevalence of diabetes ranged from 34.84% to 36.77%, and obesity was reported in 17.2% to 18.65% of patients. CIHD was present in approximately 37.7% to 39.84% of patients, while HF was observed in 37.3% to 38.78%. Most patients did not have CKD, although 9.62%-10.99% had end-stage renal disease (ESRD) (Table 1).

**Table 1:** Baseline Characteristics of In-hospital Cardiac Arrest Patients (2017-2019) ^P^: Pearson's chi-squared test. A p-value < 0.05 indicates a statistically significant result. CKD: chronic kidney disease; ESRD: end-stage renal disease; SD: standard deviation

Baseline Characteristics	Low-Volume (N=99,620)	Medium-Volume (N=103,515)	High-Volume (N=100,895)	P-value
Age (mean ± SD)	65.30 ± 15.92	63.14 ± 16.21	62.02 ± 16.36	-
Female, N (%)	41442 (41.60%)	41717 (40.30%)	40257 (39.90%)	0.002ᴾ
Race				<0.001ᴾ
White, N (%)	67752 (68.01%)	65359 (63.14%)	61445 (60.90%)	-
Black, N (%)	15521 (15.58%)	19306 (18.65%)	24477 (24.26%)	-
Hispanic, N (%)	9374 (9.41%)	11024 (10.65%)	9131 (9.05%)	-
Asian or Pacific Islander, N (%)	2789 (2.80%)	3695 (3.57%)	1887 (1.87%)	-
Native American, N (%)	907 (0.91%)	704 (0.68%)	535 (0.53%)	-
Other, N (%)	3297 (3.31%)	3426 (3.31%)	3410 (3.38%)	-
Diabetes mellitus, N(%)	36630 (36.77%)	37721 (36.44%)	35152 (34.84%)	0.001ᴾ
Obesity, N (%)	17135 (17.20%)	18250 (17.63%)	18817 (18.65%)	0.003ᴾ
Hypertension, N (%)	25991 (26.09%)	26531 (25.63%)	25183 (24.96%)	0.053ᴾ
Hyperlipidemia, N (%)	37049 (37.19%)	39812 (38.46%)	36988 (36.66%)	0.017ᴾ
CKD				<0.001ᴾ
No CKD, N (%)	70591 (70.86%)	73610 (71.11%)	72231 (71.59%)	-
Unspecified CKD, N (%)	6276 (6.30%)	5921 (5.72%)	5680 (5.63%)	-
Stage 3 CKD, N (%)	9314 (9.35%)	9513 (9.19%)	8687 (8.61%)	-
Stage 4 CKD, N (%)	3407 (3.42%)	2940 (2.84%)	2815 (2.79%)	-
Stage 5 CKD, N (%)	448 (0.45%)	466 (0.45%)	383 (0.38%)	-
ESRD, N (%)	9583 (9.62%)	11055 (10.68%)	11088 (10.99%)	-
Chronic ischemic heart disease, N (%)	37557 (37.70%)	41240 (39.84%)	39753 (39.40%)	<0.001ᴾ
Heart failure, N (%)	37158 (37.30%)	39149 (37.82%)	39127 (38.78%)	0.028ᴾ
Cardiomyopathy, N (%)	10101 (10.14%)	11708 (11.31%)	11280 (11.18%)	<0.001ᴾ
Chronic lung disease, N (%)	20512 (20.59%)	20765 (20.06%)	19987 (19.81%)	0.212ᴾ
Transfer-in, N (%)	11217 (11.26%)	15983 (15.44%)	22025 (21.83%)	<0.001ᴾ
Insurance				<0.001ᴾ
Medicare, N (%)	58875 (59.10%)	57130 (55.19%)	53505 (53.03%)	-
Medicaid, N (%)	13887 (13.94%)	15755 (15.22%)	15245 (15.11%)	-
Private insurance, N (%)	19217 (19.29%)	21262 (20.54%)	21894 (21.70%)	-
Self-pay, N (%)	4533 (4.55%)	5818 (5.62%)	6760 (6.70%)	-
No charge, N (%)	249 (0.25%)	362 (0.35%)	414 (0.41%)	-
Other, N (%)	2859 (2.87%)	3188 (3.08%)	3097 (3.07%)	-
Median household income national quartile for the patient's ZIP code				<0.001ᴾ
First quarter, N (%)	30952 (31.07%)	33632 (32.49%)	39954 (39.60%)	-
Second quarter, N (%)	26788 (26.89%)	26800 (25.89%)	26344 (26.11%)	-
Third quarter, N (%)	22923 (23.01%)	24119 (23.30%)	21279 (21.09%)	-
Fourth quarter, N (%)	18968 (19.04%)	18964 (18.32%)	13318 (13.20%)	-
Teaching hospital, N (%)	51055 (51.25%)	80172 (77.45%)	95487 (94.64%)	<0.001ᴾ
Urban hospital, N (%)	84996 (85.32%)	99654 (96.27%)	100744 (99.85%)	<0.001ᴾ
Region				<0.001ᴾ
Northeast, N (%)	17503 (17.57%)	15351 (14.83%)	13389 (13.27%)	-
Midwest, N (%)	22703 (22.79%)	21314 (20.59%)	18968 (18.80%)	-
South, N(%)	36979 (37.12%)	41137 (39.74%)	53404 (52.93%)	-
West, N (%)	22434 (22.52%)	25713 (24.84%)	15144 (15.01%)	-
Bed size				<0.001ᴾ
Small, N (%)	36421 (36.56%)	12484 (12.06%)	1766 (1.75%)	-
Medium, N (%)	35584 (35.72%)	38197 (36.90%)	18000 (17.84%)	-
Large, N (%)	27625 (27.73%)	52834 (51.04%)	81140 (80.42%)	-
Elixhauser Comorbidity Index (mean ± SD)	5.20 ± 2.29	5.33 ± 2.33	5.31 ± 2.33	-

During the three-year pre-pandemic period, unadjusted in-hospital mortality rates were 68.49% in low-volume hospitals and 63.69% in both medium- and high-volume hospitals. After adjusting for confounding variables, the adjusted ORs for in-hospital mortality were 0.955 (95% CI: 0.899-1.014, p = 0.135) for medium-volume hospitals and 1.051 (95% CI: 0.972-1.136, p = 0.212) for high-volume hospitals, compared to low-volume hospitals. In contrast, when comparing high-volume to medium-volume hospitals, the OR for mortality was 1.100 (95% CI: 1.035-1.169, p = 0.002).

After propensity score matching, the adjusted results were as follows: *medium vs. low-volume hospitals:* OR 0.956 (95% CI: 0.892-1.025, p = 0.208); *high vs. low-volume hospitals: *OR 1.056 (95% CI: 0.934-1.193, p = 0.384); and *high vs. medium-volume hospitals:* OR 1.075 (95% CI: 1.001-1.154, p = 0.046).

In our multivariable analysis, several demographic and socioeconomic variables were found to be significantly associated with in-hospital mortality among IHCA patients. Compared to White patients, Black (OR 1.355, p < 0.001), Hispanic (OR 1.176, p < 0.001), and Asian patients (OR 1.173, p = 0.01) had higher odds of in-hospital mortality. Higher income was associated with better survival: the second income quartile (OR 0.923, p = 0.02), the third quartile (OR 0.843, p < 0.001), and the fourth quartile (OR 0.809, p < 0.001) all showed progressively lower mortality compared to the lowest quartile. Insurance status was also a significant factor. Compared to Medicare beneficiaries, patients with Medicaid (OR 0.802, p < 0.01) or private insurance (OR 0.661, p < 0.01) had lower mortality. Regionally, patients in the Midwest (OR 0.884, p = 0.01) and South (OR 0.872, p < 0.001) had lower mortality compared to those in the Northeast. In terms of hospital characteristics, private non-profit hospitals were associated with lower mortality compared to government hospitals (OR 0.857, p < 0.001). Patients with certain cardiovascular comorbidities, such as HF (OR 0.703), CIHD (OR 0.453), and cardiomyopathy (OR 0.468), had lower mortality (p < 0.001 for all).

Among IHCA patients in 2020-2021, the mean age varied by hospital volume group, with patients in high-volume hospitals having a mean age of 61.89 years, those in medium-volume hospitals having a mean age of 63.04 years, and those in low-volume hospitals having a mean age of 64.28 years. White patients comprised the largest racial group in all volume categories. The prevalence of diabetes ranged from 39.26% to 40.01% across groups. Most patients did not have CKD, and the proportion with ESRD ranged from 8.75% to 10.59%. Transfer rates varied by hospital volume group: 19.62% of IHCA patients in high-volume hospitals were transfers, while 12.18% of patients in low-volume hospitals were transferred admissions (Table 2).

**Table 2:** Baseline Characteristics of In-hospital Cardiac Arrest Patients (2020-2021) ^P^: Pearson's chi-squared test. A p-value < 0.05 indicates a statistically significant result. CKD: chronic kidney disease; ESRD: end-stage renal disease; SD: standard deviation

Baseline Characteristics	Low-Volume	Medium-Volume	High-Volume	P-value
	N=93,720	N=101,675	N=92,300	
Age (mean ± SD)	64.28 ± 15.64	63.04 ± 15.81	61.89 ± 16.01	-
Female, N (%)	37319 (39.82%)	39968 (39.31%)	35674 (38.65%)	0.084ᴾ
Race				<0.001ᴾ
White, N (%)	60103 (64.13%)	56653 (55.72%)	50119 (54.30%)	-
Black, N (%)	15914 (16.98%)	20772 (20.43%)	23730 (25.71%)	-
Hispanic, N (%)	10422 (11.12%)	15902 (15.64%)	12461 (13.50%)	-
Asian or Pacific Islander, N (%)	2924 (3.12%)	3528 (3.47%)	2261 (2.45%)	-
Native American, N (%)	984 (1.05%)	722 (0.71%)	554 (0.60%)	-
Other, N (%)	3383 (3.61%)	4087 (4.02%)	3166 (3.43%)	-
Diabetes mellitus, N (%)	36804 (39.27%)	41758 (41.07%)	36929 (40.01%)	0.017ᴾ
Obesity, N (%)	21687 (23.14%)	24514 (24.11%)	22447 (24.32%)	0.083ᴾ
Hypertension, N (%)	25004 (26.68%)	26232 (25.80%)	23407 (25.36%)	0.026ᴾ
Hyperlipidemia, N (%)	37019 (39.50%)	40812 (40.14%)	36422 (39.46%)	0.564ᴾ
Chronic kidney disease				<0.001ᴾ
No CKD, N (%)	67778 (72.32%)	72423 (71.23%)	66631 (72.19%)	-
Unspecified CKD, N (%)	5417 (5.78%)	6101 (6.00%)	5307 (5.75%)	-
Stage 3 CKD, N (%)	8978 (9.58%)	9039 (8.89%)	7753 (8.40%)	-
Stage 4 CKD, N (%)	2990 (3.19%)	2857 (2.81%)	2474 (2.68%)	-
Stage 5 CKD, N (%)	356 (0.38%)	427 (0.42%)	369 (0.40%)	-
ESRD, N (%)	8201 (8.75%)	10828 (10.65%)	9775 (10.59%)	-
Chronic ischemic heart disease, N (%)	31415 (33.52%)	35718 (35.13%)	32471 (35.18%)	0.020ᴾ
Heart failure, N (%)	32943 (35.15%)	37823 (37.20%)	35342 (38.29%)	<0.001ᴾ
Cardiomyopathy, N (%)	8932 (9.53%)	10605 (10.43%)	10421 (11.29%)	<0.001ᴾ
Chronic lung disease, N (%)	16176 (17.26%)	16736 (16.46%)	15543 (16.84%)	0.164ᴾ
Transfer-in, N (%)	11415 (12.18%)	15993 (15.73%)	18109 (19.62%)	<0.001ᴾ
Insurance				<0.001ᴾ
Medicare, N (%)	51527 (54.98%)	53207 (52.33%)	46316 (50.18%)	-
Medicaid, N (%)	14180 (15.13%)	17112 (16.83%)	15580 (16.88%)	-
Private insurance, N (%)	20028 (21.37%)	22063 (21.70%)	20223 (21.91%)	-
Self-pay, N (%)	4302 (4.59%)	5551 (5.46%)	6221 (6.74%)	-
No charge, N (%)	216 (0.23%)	437 (0.43%)	415 (0.45%)	-
Other, N (%)	3458 (3.69%)	3315 (3.26%)	3554 (3.85%)	-
Median household income national quartile for the patient's ZIP code				<0.001ᴾ
First quarter, N (%)	30131 (32.15%)	34437 (33.87%)	38628 (41.85%)	-
Second quarter, N (%)	25960 (27.70%)	26202 (25.77%)	23703 (25.68%)	-
Third quarter, N (%)	20281 (21.64%)	23497 (23.11%)	18672 (20.23%)	-
Fourth quarter, N (%)	17348 (18.51%)	17539 (17.25%)	11298 (12.24%)	-
Teaching hospital, N (%)	50075 (53.43%)	80272 (78.95%)	86227 (93.42%)	<0.001ᴾ
Urban hospital, N (%)	78547 (83.81%)	98452 (96.83%)	91931 (99.60%)	<0.001ᴾ
Region				<0.001ᴾ
Northeast, N (%)	16588 (17.70%)	15160 (14.91%)	11214 (12.15%)	-
Midwest, N (%)	20440 (21.81%)	18779 (18.47%)	14307 (15.50%)	-
South, N (%)	36588 (39.04%)	43476 (42.76%)	51393 (55.68%)	-
West, N (%)	20103 (21.45%)	24260 (23.86%)	15386 (16.67%)	-
Bed size, N (%)				<0.001ᴾ
Small, N (%)	36851 (39.32%)	16776 (16.50%)	4486 (4.86%)	-
Medium, N (%)	31855 (33.99%)	34214 (33.65%)	20528 (22.24%)	-
Large, N (%)	25014 (26.69%)	50685 (49.85%)	67287 (72.90%)	-
Elixhauser comorbidity index (mean ± SD)	5.24 ± 2.26	5.42 ± 2.26	5.47 ± 2.28	-

Before stratifying by COVID-19 status, the in-hospital mortality rates for low-, medium-, and high-volume hospitals were 72.53%, 69.65%, and 63.34%, respectively. After adjustment for confounding variables, both medium- and high-volume hospitals were associated with significantly higher in-hospital mortality compared to low-volume hospitals. The ORs were 1.109 (95% CI: 1.031-1.193, p = 0.005) for medium-volume and 1.235 (95% CI: 1.131-1.349, p < 0.001) for high-volume hospitals. Furthermore, high-volume hospitals exhibited significantly higher mortality than medium-volume hospitals, with an OR of 1.114 (95% CI: 1.034-1.200, p = 0.001). Factors independently associated with lower mortality included younger age, female sex, White race, higher income, private insurance coverage, larger hospital bed size, teaching hospital status, urban hospital location, and a history of chronic ischemic heart disease (Table 3).

**Table 3:** Factors Associated With In-Hospital Mortality Among In-Hospital Cardiac Arrest Patients ᴸ: From multivariate logistic regression. A p-value < 0.05 indicates a statistically significant result. OR: odds ratio; CI: confidence interval

Factors	OR	P-value	95% CI
Female vs. male	0.925	<0.001ᴸ	0.889–0.963
Black vs. White	1.509	<0.001ᴸ	1.419–1.605
Hispanic vs. White	1.671	<0.001ᴸ	1.551–1.800
Asian or Pacific Islander vs. White	1.397	<0.001ᴸ	1.233–1.583
Native American vs. White	1.211	0.116ᴸ	0.954–1.537
2nd quartile vs. 1st quartile income	0.892	<0.001ᴸ	0.843–0.945
3rd quartile vs. 1st quartile income	0.798	<0.001ᴸ	0.749–0.850
4th quartile vs. 1st quartile income	0.742	<0.001ᴸ	0.691–0.798
Private non-profit vs. government, non-federal	0.826	<0.001ᴸ	0.745–0.915
Urban vs. rural hospitals	0.672	<0.001ᴸ	0.592–0.762
Teaching vs. non-teaching hospitals	0.701	<0.001ᴸ	0.649–0.757
Medium vs. small bed-size hospitals	0.803	<0.001ᴸ	0.742–0.870
Large vs. small bed-size hospitals	0.568	<0.001ᴸ	0.520–0.620
Chronic ischemic heart disease	0.404	<0.001ᴸ	0.385–0.424
Heart failure	0.623	<0.001ᴸ	0.589–0.659
Cardiomyopathy	0.483	<0.001ᴸ	0.452–0.514

**Figure 1:**
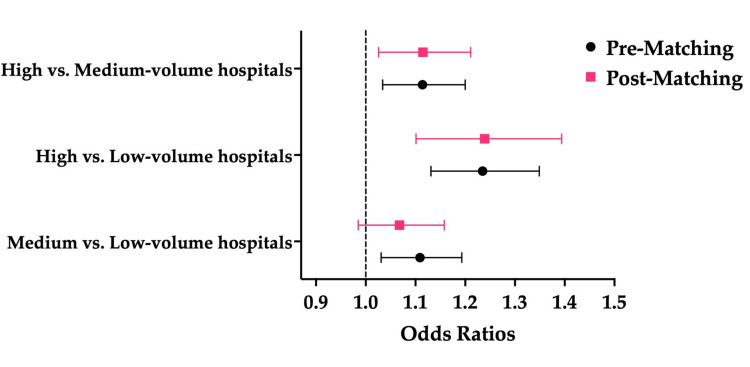
Adjusted Odds Ratios for In-Hospital Mortality Before and After Propensity Score Matching (2020-2021) Pre-matching: before propensity score matching; post-matching: after propensity score matching.

After propensity score matching, in pairwise comparisons between hospital volume groups, no statistically significant difference in in-hospital mortality was observed between medium- and low-volume hospitals. However, high-volume hospitals demonstrated significantly higher mortality compared to both low-volume hospitals (OR: 1.239; 95% CI: 1.101-1.394, p < 0.001) and medium-volume hospitals (OR: 1.115; 95% CI: 1.026-1.211, p = 0.001) (Figure 1).

We then stratified IHCA patients from 2020 to 2021 into two groups: COVID and non-COVID. Racial differences were observed between the subgroups. Descriptive characteristics of these subgroups are presented in Tables 4, 5.

**Table 4:** Baseline Characteristics of Non-COVID-19 In-Hospital Cardiac Arrest Patients ^P^: Pearson's chi-squared test. A p-value < 0.05 indicates a statistically significant result. CKD: chronic kidney disease; ESRD: end-stage renal disease; SD: standard deviation

Baseline Characteristics	Low-Volume	Medium-Volume	High-Volume	P-value
	N=72,975	N=80,690	N=73,710	
Age (mean ± SD)	64.39 ± 15.96	63.15 ± 16.05	61.88 ± 16.20	-
Female, N (%)	29642 (40.62%)	32195 (39.90%)	28644 (38.86%)	0.011ᴾ
Race				<0.001ᴾ
White, N (%)	49608 (67.98%)	48874 (60.57%)	42826 (58.10%)	-
Black, N (%)	11654 (15.97%)	15864 (19.66%)	18214 (24.71%)	-
Hispanic, N (%)	6225 (8.53%)	9723 (12.05%)	8197 (11.12%)	-
Asian or Pacific Islander, N (%)	2299 (3.15%)	2687 (3.33%)	1651 (2.24%)	-
Native American, N (%)	759 (1.04%)	565 (0.70%)	428 (0.58%)	-
Other, N (%)	2430 (3.33%)	2961 (3.67%)	2403 (3.26%)	-
Diabetes mellitus, N (%)	26512 (36.33%)	30759 (38.12%)	27206 (36.91%)	0.022ᴾ
Obesity, N (%)	14376 (19.70%)	16501 (20.45%)	15376 (20.86%)	0.109ᴾ
Hypertension, N (%)	17689 (24.24%)	19325 (23.95%)	17278 (23.44%)	0.300ᴾ
Hyperlipidemia, N (%)	28956 (39.68%)	32647 (40.46%)	29285 (39.73%)	0.534ᴾ
Chronic kidney disease				<0.001ᴾ
No CKD, N (%)	52440 (71.86%)	57225 (70.92%)	53115 (72.06%)	-
Unspecified CKD, N (%)	4108 (5.63%)	4688 (5.81%)	4061 (5.51%)	-
Stage 3 CKD, N (%)	6925 (9.49%)	7133 (8.84%)	6118 (8.30%)	-
Stage 4 CKD, N (%)	2415 (3.31%)	2364 (2.93%)	2042 (2.77%)	-
Stage 5 CKD, N (%)	285 (0.39%)	339 (0.42%)	310 (0.42%)	-
ESRD, N (%)	6801 (9.32%)	8949 (11.09%)	8064 (10.94%)	-
Chronic ischemic heart disease, N (%)	26928 (36.90%)	31372 (38.88%)	28364 (38.48%)	0.019ᴾ
Heart failure, N (%)	28497 (39.05%)	32760 (40.60%)	30391 (41.23%)	0.004ᴾ
Cardiomyopathy, N (%)	7940 (10.88%)	9433 (11.69%)	9192 (12.47%)	<0.001ᴾ
Chronic lung disease, N (%)	14099 (19.32%)	14807 (18.35%)	13857 (18.80%)	0.140ᴾ
Transfer-in, N (%)	9012 (12.35%)	13419 (16.63%)	15052 (20.42%)	<0.001ᴾ
Insurance				<0.001ᴾ
Medicare, N (%)	41253 (56.53%)	43072 (53.38%)	37585 (50.99%)	-
Medicaid, N (%)	11267 (15.44%)	13459 (16.68%)	12509 (16.97%)	-
Private insurance, N (%)	14515 (19.89%)	16638 (20.62%)	15361 (20.84%)	-
Self-pay, N (%)	3430 (4.70%)	4575 (5.67%)	5292 (7.18%)	-
No charge, N (%)	168 (0.23%)	363 (0.45%)	361 (0.49%)	-
Other, N (%)	2335 (3.20%)	2574 (3.19%)	2602 (3.53%)	-
Median household income national quartile for patient's ZIP code				<0.001ᴾ
First quarter, N (%)	22476 (30.80%)	26111 (32.36%)	29985 (40.68%)	-
Second quarter, N (%)	20221 (27.71%)	20955 (25.97%)	19106 (25.92%)	-
Third quarter, N (%)	15967 (21.88%)	18938 (23.47%)	15096 (20.48%)	-
Fourth quarter, N (%)	14310 (19.61%)	14686 (18.20%)	9523 (12.92%)	-
Teaching hospital, N (%)	40224 (55.12%)	64851 (80.37%)	69192 (93.87%)	<0.001ᴾ
Urban hospital, N (%)	62058 (85.04%)	78148 (96.85%)	73437 (99.63%)	<0.001ᴾ
Region				<0.001ᴾ
Northeast, N (%)	13033 (17.86%)	11902 (14.75%)	9125 (12.38%)	-
Midwest, N (%)	16719 (22.91%)	16041 (19.88%)	12597 (17.09%)	-
South, N (%)	27351 (37.48%)	33728 (41.80%)	40194 (54.53%)	-
West, N (%)	15872 (21.75%)	19019 (23.57%)	11794 (16.00%)	-
Bed size				<0.001ᴾ
Small, N (%)	28073 (38.47%)	12338 (15.29%)	2985 (4.05%)	-
Medium, N (%)	24848 (34.05%)	26579 (32.94%)	15118 (20.51%)	-
Large, N (%)	20061 (27.49%)	41773 (51.77%)	55607 (75.44%)	-
Elixhauser comorbidity index (mean ± SD)	5.35 ± 2.29	5.49 ± 2.29	5.52 ± 2.34	-

**Table 5:** Baseline Characteristics of COVID-19 In-Hospital Cardiac Arrest Patients ^P^: Pearson's chi-squared test. A p-value < 0.05 indicates a statistically significant result. CKD: chronic kidney disease; ESRD: end-stage renal disease; SD: standard deviation

Baseline Characteristics	Low-Volume	Medium-Volume	High-Volume	P-value
	N=20745	N=20985	N=18590	
Age (mean ± SD)	63.91 ± 14.47	62.62 ± 14.84	61.91 ± 15.26	-
Female, N (%)	7676 (37.00%)	7771 (37.03%)	7033 (37.83%)	0.698ᴾ
Race				<0.001ᴾ
White, N (%)	10514 (50.68%)	7809 (37.21%)	7310 (39.32%)	-
Black, N (%)	4255 (20.51%)	4904 (23.37%)	5518 (29.68%)	-
Hispanic, N (%)	4182 (20.16%)	6157 (29.34%)	4259 (22.91%)	-
Asian or Pacific Islander, N (%)	624 (3.01%)	841 (4.01%)	613 (3.30%)	-
Native American, N (%)	222 (1.07%)	153 (0.73%)	128 (0.69%)	-
Other, N (%)	948 (4.57%)	1119 (5.33%)	762 (4.10%)	-
Diabetes mellitus, N (%)	10285 (49.58%)	11000 (52.42%)	9721 (52.29%)	0.021ᴾ
Obesity, N (%)	7311 (35.24%)	8010 (38.17%)	7075 (38.06%)	0.027ᴾ
Hypertension, N (%)	7315 (35.26%)	6904 (32.90%)	6129 (32.97%)	0.052ᴾ
Hyperlipidemia, N (%)	8059 (38.85%)	8159 (38.88%)	7135 (38.38%)	0.903ᴾ
Chronic kidney disease				0.010ᴾ
No CKD, N (%)	15341 (73.95%)	15199 (72.43%)	13515 (72.70%)	-
Unspecified CKD, N (%)	1309 (6.31%)	1414 (6.74%)	1249 (6.72%)	-
Stage 3 CKD, N (%)	2050 (9.88%)	1910 (9.10%)	1636 (8.80%)	-
Stage 4 CKD, N (%)	575 (2.77%)	491 (2.34%)	426 (2.29%)	-
Stage 5 CKD, N (%)	71 (0.34%)	86 (0.41%)	56 (0.30%)	-
ESRD, N (%)	1400 (6.75%)	1884 (8.98%)	1710 (9.20%)	-
Chronic ischemic heart disease, N (%)	4489 (21.64%)	4346 (20.71%)	4110 (22.11%)	0.367ᴾ
Heart failure, N (%)	4450 (21.45%)	5059 (24.11%)	4954 (26.65%)	<0.001ᴾ
Cardiomyopathy, N (%)	990 (4.77%)	1165 (5.55%)	1231 (6.62%)	0.003ᴾ
Chronic lung disease, N (%)	2081 (10.03%)	1931 (9.20%)	1681 (9.04%)	0.287ᴾ
Transfer-in, N (%)	2402 (11.58%)	2577 (12.28%)	3060 (16.46%)	<0.001ᴾ
Insurance				0.001ᴾ
Medicare, N (%)	10269 (49.50%)	10129 (48.27%)	8735 (46.99%)	-
Medicaid, N (%)	2917 (14.06%)	3645 (17.37%)	3073 (16.53%)	-
Private insurance, N (%)	5516 (26.59%)	5427 (25.86%)	4858 (26.13%)	-
Self-pay, N (%)	873 (4.21%)	976 (4.65%)	922 (4.96%)	-
No charge, N (%)	46 (0.22%)	69 (0.33%)	50 (0.27%)	-
Other, N (%)	1124 (5.42%)	737 (3.51%)	952 (5.12%)	-
Median household income national quartile for patient's ZIP code				<0.001ᴾ
First quarter, N (%)	7651 (36.88%)	8323 (39.66%)	8642 (46.49%)	-
Second quarter, N (%)	5734 (27.64%)	5250 (25.02%)	4597 (24.73%)	-
Third quarter, N (%)	4315 (20.80%)	4560 (21.73%)	3573 (19.22%)	-
Fourth quarter, N (%)	3045 (14.68%)	2852 (13.59%)	1777 (9.56%)	-
Teaching hospital, N (%)	9850 (47.48%)	15420 (73.48%)	17030 (91.61%)	<0.001ᴾ
Urban hospital, N (%)	16484 (79.46%)	20301 (96.74%)	18490 (99.46%)	<0.001ᴾ
Region				<0.001ᴾ
Northeast, N (%)	3556 (17.14%)	3259 (15.53%)	2090 (11.24%)	-
Midwest, N (%)	3720 (17.93%)	2734 (13.03%)	1710 (9.20%)	-
South, N (%)	9236 (44.52%)	9750 (46.46%)	11195 (60.22%)	-
West, N (%)	4234 (20.41%)	5240 (24.97%)	3595 (19.34%)	-
Bed size				<0.001ᴾ
Small, N (%)	8779 (42.32%)	4445 (21.18%)	1500 (8.07%)	-
Medium, N (%)	7016 (33.82%)	7630 (36.36%)	5410 (29.10%)	-
Large, N (%)	4950 (23.86%)	8910 (42.46%)	11680 (62.83%)	-
Elixhauser comorbidity index, N (%)	4.83 ± 2.10	5.13 ± 2.13	5.27 ± 2.05	-

In the COVID-positive group, interestingly, no significant association was found between hospital volume and mortality. After propensity matching, the following results were observed:​​​​​​​ *medium vs. low-volume hospitals: *OR 1.125 (95% CI: 0.856-1.480, p = 0.39); ​​​​​​​*high vs. low-volume hospitals:* OR 1.387 (95% CI: 0.942-2.042, p = 0.098); and ​​​​​​​*high vs. medium-volume hospitals:* OR 1.116 (95% CI: 0.883-1.411, p = 0.357).

**Figure 2:**
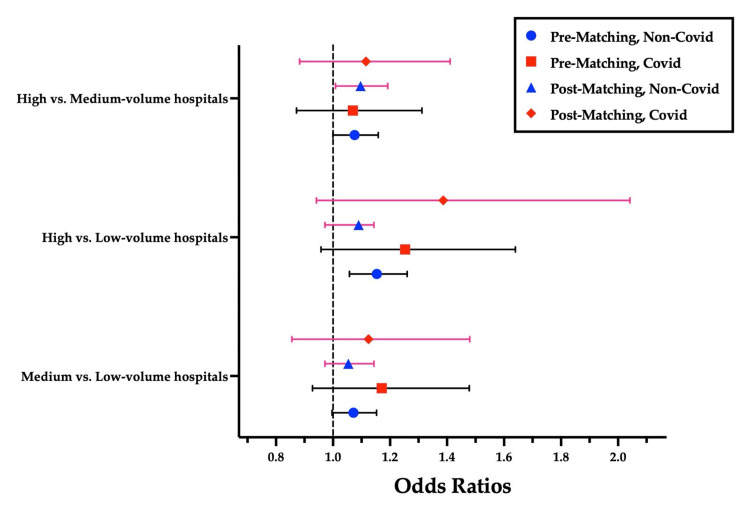
Adjusted Odds Ratios for In-Hospital Mortality Before and After Propensity Score Matching in COVID-Positive and Non-COVID In-Hospital Cardiac Arrest Patients Pre-matching: before propensity score matching; post-matching: after propensity score matching.

In the non-COVID group, prior to propensity score matching, high-volume hospitals were associated with significantly higher in-hospital mortality compared to low-volume hospitals (OR: 1.154; 95% CI: 1.058-1.260, p = 0.001) and medium-volume hospitals (OR: 1.076; 95% CI: 1.000-1.159, p = 0.049). After matching, the ORs for mortality were as follows:​​​​​​​ *medium vs. low-volume hospitals: *OR 1.054 (95% CI: 0.972-1.144, p = 0.203); ​​​​​​​*high vs. low-volume hospitals:* OR 1.090 (95% CI: 0.972-1.144, p = 0.157); and ​​​​​​​*high vs. medium-volume hospitals:* OR 1.097 (95% CI: 1.009-1.192, p = 0.003) (Figure 2). The matched groups showed adequate balance in baseline characteristics and sample sizes across all pairwise comparisons.

## Discussion

In this large, nationally representative analysis of over 500,000 patients with IHCA from 2017 to 2021, we found that hospital volume was significantly associated with mortality, but the direction and strength of this association varied by time period and COVID-19 status.

Pre-pandemic volume-outcome relationship

From 2017 to 2019, before propensity score matching, high-volume hospitals showed a significantly higher mortality rate compared to medium-volume hospitals. After propensity score matching, high-volume hospitals continued to demonstrate modestly higher odds of in-hospital mortality compared to medium-volume centers (OR: 1.075, 95% CI: 1.001-1.154, p = 0.046). However, the confidence interval barely excludes unity, and the p-value lies just below the conventional cutoff, suggesting that the clinical significance of this difference may be limited. Cautious interpretation is therefore warranted.

The absence of a clear volume-mortality association between low- and high-volume hospitals during this period highlights the protocolized nature of the IHCA response. Standardized code blue protocols, rapid response systems, and post-arrest care bundles may have helped mitigate outcome differences across institutions, particularly in hospitals with fewer overall IHCA cases. The higher mortality observed in high-volume hospitals compared to medium-volume centers suggests that increasing volume alone does not guarantee better outcomes. This trend may reflect operational strain, more complex case mixes, or the impact of interhospital transfers. In our cohort, transfer-in rates were highest in high-volume hospitals (28.31%), followed by medium-volume (15.44%) and low-volume hospitals (11.26%). A multicenter analysis has shown that patients transferred into tertiary medical ICUs had a 25% in-hospital mortality rate, compared with 21% for direct admissions, even after adjustment [13]. Medium-volume hospitals may strike a balance between sufficient case volume to maintain proficiency and manageable workloads that prevent care fragmentation or delays.

Pandemic-era volume-outcome relationship

During 2020-2021, we observed a significant increase in in-hospital mortality among IHCA patients, with 56,470 deaths (93.62%), consistent with prior findings from the GWTG (Get With The Guidelines) registry [14].

In-hospital mortality for IHCA rose markedly, particularly in high-volume hospitals. Compared to low- and medium-volume hospitals, the adjusted ORs after propensity score matching were 1.239 (95% CI: 1.101-1.394, p < 0.001) and 1.115 (95% CI: 1.026-1.211, p = 0.001), respectively. This decline in IHCA survival during the pandemic appears to have been driven in part by system-level stress, particularly in referral centers that managed both COVID-19 and non-COVID critically ill patients. These high-volume hospitals encountered several key challenges:

ICU Overcrowding and Staffing Shortages

The rapid rise in ICU census strained nurse-to-patient ratios and reduced intensivist availability. The World Health Organization (WHO) reported a global shortfall of approximately 13 million nursing and midwifery professionals in 2020 [15]. Staffing shortages can severely limit the ability to deliver timely, high-quality CPR and post-arrest care [16].

Operational Delays

Strict infection control measures, including the donning and doffing of personal protective equipment and the use of negative-pressure rooms, delayed code team activation and defibrillation. While the extent of these delays remains debated, such protocols likely contributed to slower response times [1].

Resource Diversion

During peak surges, access to essential post-resuscitation interventions such as catheterization labs and targeted temperature management was limited [17]. A cohort study published in JAMA Network Open found that hospitals with the highest IHCA survival rates typically excelled in either acute resuscitation or post-resuscitation care [9].

Higher Comorbidity Burden in Vulnerable Populations

High-volume hospitals often serve socioeconomically disadvantaged populations with more complex medical needs. We found that these centers cared for a larger proportion of patients with multiple comorbidities, which likely intensified resource strain and further contributed to poorer outcomes during the pandemic.

Role of SARS-CoV-2 infection

When stratified by COVID-19 status, we found that among COVID-positive IHCA patients, who experience extremely high mortality rates (87%-90%) [14,18], hospital volume was not significantly associated with outcomes. This finding remained consistent even after propensity score matching between hospital volume groups. In contrast, within the non-COVID group, IHCA mortality was significantly higher among patients admitted to high-volume hospitals. These findings suggest that SARS-CoV-2 infection alone does not modify the relationship between hospital volume and IHCA mortality. Instead, the increased mortality observed in high-volume hospitals during 2020-2021 is more likely attributable to system-level burdens imposed by the pandemic.

Other factors associated with in-hospital mortality

Several hospital-level characteristics were significantly associated with improved survival following IHCA. These included teaching hospital status, urban location, larger bed capacity, and private nonprofit ownership.

At the patient level, factors associated with lower in-hospital mortality included younger age, female sex, White race, higher income, private insurance coverage, hypertension, CIHD, HF, and cardiomyopathy. Interestingly, patients with certain chronic cardiac conditions demonstrated lower mortality, which may be explained by several possible mechanisms:

Secondary Prevention Effect

Patients with pre-existing cardiovascular disease may be more likely to be on cardioprotective medications, such as statins and beta-blockers. Statin therapy has been independently associated with lower long-term mortality in patients presenting with ventricular tachyarrhythmias [19]. Additionally, prior use of high-dose beta-blockers before cardiac arrest has been linked to improved one-year survival [20].

Healthy-User Effect

Individuals who adhere to preventive medications are often more engaged in health-promoting behaviors overall. This may contribute to better outcomes, independent of the medication effects [21].

Residual Confounding

Administrative databases, such as the NIS, lack detailed clinical variables and may not fully capture illness severity. This limitation introduces the potential for residual confounding, where unmeasured factors may influence the observed associations [22].

Strengths and limitations

The strengths of this study include the use of a large, nationally representative sample drawn from the NIS database, robust adjustment for patient and hospital-level risk factors, and the application of propensity score matching to reduce confounding between hospital volume groups.

However, several limitations must be acknowledged. As with all studies using administrative data, there is a potential for coding inaccuracies and misclassification of clinical conditions. We identified IHCA cases using a secondary diagnosis of cardiac arrest, based on the rationale that events occurring during hospitalization are more likely to be recorded in secondary diagnosis fields, while OHCA is typically coded as a principal diagnosis at admission. However, this approach may misclassify some cases, particularly in complex admissions where IHCA may be listed as the principal diagnosis. This reflects a fundamental limitation of the NIS, which lacks clinical detail such as the timing and setting of cardiac arrest events.

Although the NIS contains discharge-level data that includes the month and quarter of admission, allowing for stratification by specific pandemic phases (e.g., initial surge vs. later waves), we did not perform this level of temporal granularity in the current analysis due to the complexity and volume of the data. As a result, we pooled all admissions from 2020 and 2021 to ensure statistical power and model stability. However, we acknowledge that different phases of the pandemic may have had varying impacts on IHCA outcomes due to evolving clinical practices, hospital strain, and resource allocation. Future studies may benefit from examining these temporal patterns in more detail.

We also acknowledge the possibility of selection bias at the hospital level. High-volume hospitals often act as referral centers, receiving more complex or critically ill patients transferred from other facilities. This may partly explain the higher adjusted mortality rates observed, despite the lower unadjusted mortality rates. Although we adjusted for numerous patient and hospital characteristics, including transfer status and comorbidity burden, residual confounding remains a possibility. Moreover, the NIS does not capture granular clinical information, such as initial arrest rhythm, timing of interventions, resuscitation quality, ROSC (return of spontaneous circulation) data, or use of targeted post-arrest therapies, all of which are critical determinants of cardiac arrest outcomes.

## Conclusions

In this large retrospective analysis of over 500,000 IHCA cases from 2017 to 2021, hospital volume was associated with in-hospital mortality, although this relationship varied by pandemic period and patient subgroup. In the pre-pandemic era (2017-2019), higher volume correlated with increased mortality overall and persisted among non-COVID-19 patients, whereas no significant volume-outcome relationship was observed in COVID-positive patients. These patterns suggest that system-level pressures, such as staffing shortages, ICU capacity constraints, and interfacility transfers, rather than direct viral pathophysiology, likely drove the volume-mortality associations seen during the pandemic.

Our findings highlight the need for surge-adaptive protocols to maintain the quality of resuscitation and post-arrest care under operational stress. Potential strategies include cross-trained resuscitation teams, scalable ICU staffing models, telemedicine support, and dynamic hospital strain surveillance systems.

## References

[1] Andersen LW, Holmberg MJ, Berg KM, Donnino MW, Granfeldt A (2019). In-hospital cardiac arrest: a review. JAMA.

[2] Tsai JC, Ma JW, Liu SC, Lin TC, Hu SY (2021). Cardiac arrest survival postresuscitation in-hospital (CASPRI) score predicts neurological favorable survival in emergency department cardiac arrest. J Clin Med.

[3] Chan PS, Kennedy KF, Girotra S (2023). Updating the model for risk-standardizing survival for in-hospital cardiac arrest to facilitate hospital comparisons. Resuscitation.

[4] Merchant RM, Becker LB, Brooks SC (2024). The American Heart Association Emergency Cardiovascular Care 2030 impact goals and call to action to improve cardiac arrest outcomes: a scientific statement from the American Heart Association. Circulation.

[5] Nallamothu BK, Guetterman TC, Harrod M (2018). How do resuscitation teams at top-performing hospitals for in-hospital cardiac arrest succeed? A qualitative study. Circulation.

[6] Lee S, Lee SW, Han KS, Ki M, Ko YH, Kim SJ (2021). Analysis of characteristics and mortality in cardiac arrest patients by hospital level: a nationwide population-based study. J Korean Med Sci.

[7] Akintoye E, Adegbala O, Egbe A, Olawusi E, Afonso L, Briasoulis A (2020). Association between hospital volume of cardiopulmonary resuscitation for in-hospital cardiac arrest and survival to hospital discharge. Resuscitation.

[8] (2021). Healthcare Cost and Utilization Project (HCUP): Overview of the National Inpatient Sample (NIS). Healthcare Research and Quality.

[9] Girotra S, Nallamothu BK, Tang Y, Chan PS (2020). Association of hospital-level acute resuscitation and postresuscitation survival with overall risk-standardized survival to discharge for in-hospital cardiac arrest. JAMA Netw Open.

[10] Sharma N, Schwendimann R, Endrich O, Ausserhofer D, Simon M (2021). Comparing Charlson and Elixhauser comorbidity indices with different weightings to predict in-hospital mortality: an analysis of national inpatient data. BMC Health Serv Res.

[11] Kontos MC, Fordyce CB, Chen AY, Chiswell K, Enriquez JR, de Lemos J, Roe MT (2019). Association of acute myocardial infarction cardiac arrest patient volume and in-hospital mortality in the United States: insights from the National Cardiovascular Data Registry Acute Coronary Treatment and Intervention Outcomes Network Registry. Clin Cardiol.

[12] (2023). The complete statistical software for data science. https://www.stata.com.

[13] Durairaj L, Will JG, Torner JC, Doebbeling BN (2003). Prognostic factors for mortality following interhospital transfers to the medical intensive care unit of a tertiary referral center. Crit Care Med.

[14] Isath A, Malik A, Bandyopadhyay D, Goel A, Rosenzveig A, Cooper HA, Panza JA (2023). Nationwide analysis of cardiac arrest outcomes during the COVID-19 pandemic. Curr Probl Cardiol.

[15] Hynes L, Geraghty S, McChlery S, Smyth A, Brar R, Clark-Burg K (2025). Nurses' and midwives' job satisfaction and retention during COVID-19: a scoping review. BMC Nurs.

[16] Janatolmakan M, Nouri R, Soroush A, Andayeshgar B, Khatony A (2021). Barriers to the success of cardiopulmonary resuscitation from the perspective of Iranian nurses: a qualitative content analysis. Int Emerg Nurs.

[17] Melman GJ, Parlikad AK, Cameron EA (2021). Balancing scarce hospital resources during the COVID-19 pandemic using discrete-event simulation. Health Care Manag Sci.

[18] Ippolito M, Catalisano G, Marino C (2021). Mortality after in-hospital cardiac arrest in patients with COVID-19: a systematic review and meta-analysis. Resuscitation.

[19] Rusnak J, Behnes M, Schupp T (2019). Statin therapy is associated with improved survival in patients with ventricular tachyarrhythmias. Lipids Health Dis.

[20] Huang HC, Yu PH, Tsai MS, Chien KL, Chen WJ, Huang CH (2021). Prior beta-blocker treatment improves outcomes in out-of-hospital cardiac arrest patients with non-shockable rhythms. Sci Rep.

[21] Olawore O, Stϋrmer T, Glynn RJ, Lund JL (2024). The healthy user effect in pharmacoepidemiology. Am J Epidemiol.

[22] Johnson EK, Nelson CP (2013). Values and pitfalls of the use of administrative databases for outcomes assessment. J Urol.

